# Conducting Molecular Epidemiological Research in the Age of HIPAA: A Multi-Institutional Case-Control Study of Breast Cancer in African-American and European-American Women

**DOI:** 10.1155/2009/871250

**Published:** 2009-10-25

**Authors:** Christine B. Ambrosone, Gregory L. Ciupak, Elisa V. Bandera, Lina Jandorf, Dana H. Bovbjerg, Gary Zirpoli, Karen Pawlish, James Godbold, Helena Furberg, Anne Fatone, Heiddis Valdimarsdottir, Song Yao, Yulin Li, Helena Hwang, Warren Davis, Michelle Roberts, Lara Sucheston, Kitaw Demissie, Kandace L. Amend, Paul Tartter, James Reilly, Benjamin W. Pace, Thomas Rohan, Joseph Sparano, George Raptis, Maria Castaldi, Alison Estabrook, Sheldon Feldman, Christina Weltz, Margaret Kemeny

**Affiliations:** ^1^Department of Cancer Prevention & Control, Roswell Park Cancer Institute, Elm & Carlton Streets, Buffalo, NY 14263, USA; ^2^Cancer Institute of New Jersey, UMDNJ's Robert Wood Johnson Medical School, 195 Little Albany Street, New Brunswick, NJ 08903-2681, USA; ^3^Mount Sinai School of Medicine, One Gustave L. Levy Place, New York, NY 10029, USA; ^4^Department of Medicine, University of Pittsburgh Cancer Institute, 5150 Centre Avenue, Pittsburgh, PA 15232, USA; ^5^Cancer Epidemiology Services, New Jersey State Department of Health and Senior Services, P. O. Box 369, Trenton, NJ 08625-0369, USA; ^6^Department of Genetics, University of North Carolina at Chapel Hill, 120 Mason Farm Road, 5000 D, GMB, CB number 7264, UNC-Chapel Hill, Chapel Hill, NC 27599-7264, USA; ^7^School of Public Health, University of Medicine and Dentistry of NJ, 683 Hoes Lane West, Building RWJ-SPH, Room 212, Piscataway, NJ 08854, USA; ^8^i3 Drug Safety, 5430 Data Court, Street number 200, Ann Arbor, MI 48108, USA; ^9^Department of Breast and Oncology Surgery, St. Luke's-Roosevelt Hospital, 1111 Amsterdam Avenue, New York, NY 10025, USA; ^10^Department of Surgery, Kings County Hospital, Kings County Hospital Center, SUNY-Downstate College of Medicine, 451 Clarkson Avenue, Brooklyn, NY11203, USA; ^11^Department of Epidemiology and Population Health, Albert Einstein College of Medicine, Jack and Pearl Resnick Campus, 1300 Morris Park Avenue, Bronx, NY 10461, USA; ^12^Departments of Medicine, Obstetrics and Gynecology, Montefiore Medical Center, Jack D. Weiler Hospital, 1825 Eastchester Road, Room 2S-47-48, Bronx, NY 10461, USA; ^13^Department of Surgery, Jacobi Medical Center, 1400 Pelham Parkway South, Bronx, NY 10461, USA; ^14^Department of Surgery, New York-Presbyterian/Columbia, 161 Fort Washington Avenue, Street 1025, New York, NY 10032, USA; ^15^Department of Surgical Oncology, Queens Cancer Center, Queens Hospital Center, 82-68 164th Street, New Bldg, 5th floor, Jamaica, New York, NY 11432, USA

## Abstract

Breast cancer in African-American (AA) women occurs at an earlier age than in European-American (EA) women and is more likely to have aggressive features associated with poorer prognosis, such as high-grade and negative estrogen receptor (ER) status. The mechanisms underlying these differences are unknown. To address this, we conducted a case-control study to evaluate risk factors for high-grade ER- disease in both AA and EA women. With the onset of the Health Insurance Portability and Accountability Act of 1996, creative measures were needed to adapt case ascertainment and contact procedures to this new environment of patient privacy. In this paper, we report on our approach to establishing a multicenter study of breast cancer in New York and New Jersey, provide preliminary distributions of demographic and pathologic characteristics among case and control participants by race, and contrast participation rates by approaches to case ascertainment, with discussion of strengths and weaknesses.

## 1. Introduction

### 1.1. Rationale for the Study

Although breast cancer incidence is higher overall in women of European descent than in women of African ancestry, African-American (AA) women are more likely than European-American (EA) women to be diagnosed before age 40 and to have breast tumors with more aggressive features, including high-grade and negative estrogen receptor (ER) status (reviewed in [1]). There are no facile explanations for these differences in the epidemiology of breast cancer by ancestry. There have been several studies of breast cancer risk that include both AA and EA women, such as the Carolina Breast Cancer Study, the CARE Study, and the Black Women's Health Study; however, none were specifically designed and powered to evaluate numerous risk factors for early/aggressive breast cancer and to evaluate the distribution of these risk factors within and across racial/ethnic groups. Because of the large, racially mixed population of women in metropolitan New York City (NYC) and eastern New Jersey (NJ), we are currently conducting a case-control study, the Women's Circle of Health Study (WCHS), with the goal of accruing 1200 AA and 1200 EA women with breast cancer and an equal number of controls, to specifically address these questions. Initial funding for this study was through a Center of Excellence for Biobehavioral Breast Cancer Research (Bovbjerg, PI) focusing on AA women, funded by the Department of Defense (DOD). Additional R01 funding (Ambrosone, PI) from the National Cancer Institute (NCI) was subsequently obtained which allowed us to increase the sample size and to extend the study to EA women. Additional facets of the study are funded by the Breast Cancer Research Foundation.

## 2. Materials and Methods

**Figure 1:**
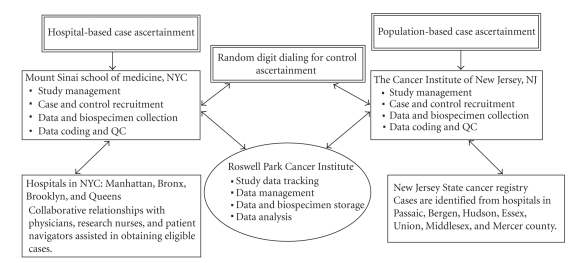
Organization and administration of the Women's Circle of Health Study.

As illustrated in Figure 1, the study has included two bases for recruitment and interviewing, one in NYC, based at Mount Sinai School of Medicine (MSSM), and one in NJ, based at The Cancer Institute of New Jersey (CINJ), with data and biospecimens sent to Roswell Park Cancer Institute (RPCI) in Buffalo, NY, for processing and storage. In the NYC metropolitan region, there are more than 60 hospitals where surgery for breast cancer is performed. When this study began in 2003, to maximize efficiency, we targeted the hospitals that had the greatest referral patterns for AA women in the boroughs of Manhattan, Brooklyn, Queens, and the Bronx. Our initial plan was to employ the approach commonly used in case-control studies, such as the Carolina Breast Cancer Study [2] and the Long Island Breast Cancer Study Project [3], wherein rapid case ascertainment is used to identify women newly diagnosed with breast cancer through periodic review of pathology reports in the targeted hospitals. When women with breast cancer are identified, a letter is sent to the treating physician, notifying them that unless they object, the patient will be contacted to describe the study and assess interest in participation.

We were unable to use this approach, however, due to the implementation of the Health Insurance Portability and Accountability Act (HIPAA) Privacy Rule in 2003, while we were establishing the infrastructure for the study. This extension of the HIPAA regulation prevents the release of private health information (PHI) without consent from the patient. For our research purposes, this Act prevented the identification of eligible cases without the patients' prior permission given to their doctors. Although there may be situations in which an HIPAA waiver can be obtained to circumvent the need to obtain patient permission for release of identifying information to researchers [4, 5], the several participating hospitals and their Institutional Review Boards (IRB), many not extensively familiar with epidemiological research, would not grant these waivers to allow patient identification. Thus, we developed a procedure for patient ascertainment and contact that complied with the regulations of HIPAA.

As an alternative strategy, we expanded our catchment area to include eastern NJ, by partnering with CINJ and the NJ State Cancer Registry, a Surveillance, Epidemiology and End Results Program (SEER) site, housed at the NJ State Department of Health and Senior Services (NJDHSS). The study has been approved by the IRB at RPCI, Robert Wood Johnson Medical School (for The CINJ), MSSM, the individual hospitals in NYC, and the NJDHSS.

In this paper we report on both of our approaches to case ascertainment and consenting, discussing effort and costs associated with each methodology. Currently, recruitment efforts are focused only in NJ, and accrual has been discontinued in NY. We also present an overview of the study design, report on distributions of demographic and selected breast cancer risk factors among both cases and controls by race/ethnicity, and compare clinical breast cancer characteristics between groups in a subset of the population enrolled to date.

### 2.1. Hospital-Based Case Ascertainment and Contact: New York City

AA and EA women, 20 to 65 years of age, with no previous history of cancer other than nonmelanoma skin cancer, diagnosed within 9 months with primary, histologically confirmed invasive breast cancer or ductal carcinoma in situ who speak English were eligible for participation in the study. They were ascertained from designated hospitals that have large referral patterns for AA women in the NYC boroughs (Manhattan, Bronx, Brooklyn, and Queens; due to few AA breast cancer patients, Staten Island was not included). To maintain comparability between cases and controls, women with breast cancer must have had a residential telephone given that controls were ascertained using random digit dialing (RDD). This eligibility criterion has now been expanded to cell phone usage, however, with RDD also covering cell phones for control ascertainment.

To address HIPAA regulations that prohibit identification of women with breast cancer using pathology reports, tumor registry data, or medical records, we worked to develop collaborative relationships with physicians, research nurses, and patient navigators at each of the participating hospitals. Our research assistants initiated frequent visits to each site, particularly on clinic days, and became well known by staff and clinic personnel. As we began working with physicians at each site, clinicians reviewed their records for retrospective ascertainment and identified women who were eligible to be in the study (e.g., had been diagnosed within the last 9 months). At each of the participating hospitals, physicians telephoned women who were not returning for followup and would not be seen at subsequent visits, asking if WCHS staff could contact them regarding the study. Those scheduled for routine followup appointments within the 9-month interval were seen and asked if they were willing to be contacted for this study. For contemporaneous recruitment, our study staff was present in the offices on breast clinic days and was informed by the physicians or research nurses at that time of patients scheduled on those days who were eligible for the study. Study materials were placed in the charts of the eligible patients as a reminder for the clinician to discuss the study. If in agreement, the patient was then referred to our waiting study staff. A number of patients participated in the informed consent procedures at the time that they were first approached and a pretreatment blood specimen was obtained. Other women preferred to be contacted at a later date by the Research Assistant (RA)/Study Interviewer, to schedule a date to obtain consent and conduct the in-person interview.

To strive for complete case ascertainment, we periodically requested that physicians review their records to confirm that we had not missed potential cases, and that they follow the procedures described above if there were women who were not previously approached to participate in the study. It was our intent that this periodic review would allow us to estimate a denominator, to some extent, and to keep track of women who refused to be contacted so that selection bias could be examined. However, these data were not easily obtained with our inability to access records of women diagnosed who had not been approached, and competing priorities of busy surgeons.

This approach to case ascertainment and contact yielded good participation rates for both AA and EA cases but was extremely labor intensive, requiring frequent communications between our research staff and clinical personnel as well as the presence of RAs at the hospitals on clinic days. Besides being costly in personnel time, this methodology required a good deal of dedication and commitment on the part of physicians, with frequent reminders from study staff for them to check their appointment ledgers and contact patients who may have been missed on clinic days. Because of all of the limitations of this approach, in 2006 we established collaboration with the New Jersey State Cancer Registry, based at the NJDHSS for rapid case ascertainment, and phased out recruitment in metropolitan New York, ending in December 2008.

### 2.2. Population-Based Case Ascertainment and Contact: New Jersey

In NJ, cases are actively being identified at all major hospitals in Passaic, Bergen, Hudson, Essex, Union, Middlesex, and Mercer Counties through rapid case ascertainment. In addition, NJDHSS study staff routinely check the New Jersey State Cancer Registry (NJSCR) database for eligible cases who reside in the target counties but are reported by hospitals outside of those seven counties or out-of-state. All AA women less than 65 years of age who are newly diagnosed with incident breast cancer are identified as potential participants. For each AA case, an EA woman with breast cancer is randomly selected, matching on age (±5 years) and county of residence. NJDHSS study staff review pathology reports of potential cases, contact doctors' offices, and hospitals to verify patients' race and demographics and check the NJSCR database for prior diagnoses of cancer. After contact with clinicians by NJDHSS staff for passive consent (e.g., contact from physician only in the event that they do not give permission to contact their patients), eligible women are telephoned by NJDHSS staff to obtain verbal consent to release names and contact information to WCHS research staff at CINJ. Patients who agree to be contacted by WCHS study staff are then telephoned by one of our interviewers, and appointments are scheduled for in-person interviews at home or at another mutually convenient location.

### 2.3. Control Eligibility and Identification: New York City and New Jersey

AA and EA women 20 to 65 years of age without a history of any cancer diagnosis other than non-melanoma skin cancer are eligible to be controls. The choice of a proper control group is a difficult issue in epidemiology today, particularly for a study that is not population-based. When planning for the WCHS, we evaluated several potential sources of control groups, weighing the strengths and weaknesses for each. While we considered using hospital controls in NYC, we felt that they would not necessarily represent the same populations from which the cases were derived. For example, many of the treating physicians at MSSM have private surgical practices; there is no indication that clinic patients from the hospital would be similar to those being treated by private physicians. Furthermore, there are well-recognized potential biases associated with the use of hospital controls [6]. In theory, the generalizability of study results is likely to be greater in studies using community controls rather than those using friend or hospital controls. Yet, in contrast to the Western European national health care records, none of the available United States (US) lists, such as that of licensed drivers, municipal tax roles, voter registration, and listed phone numbers, provide complete source population enumeration. Population coverage, access to this information, and the quality of contact information vary geographically in the US. Of NYC residents, it is estimated that only 52.1% have drivers licenses [7], only 30.2% pay residential taxes [8], and only 56.2% are registered voters [9]. These examples typify the acknowledged weaknesses of US and NYC sampling frames.

For generating a control group of adults under 65 years of age we used random digit dialing (RDD) because unlisted numbers can be reached by this method, thereby avoiding possible selection bias (NYC study found that 27% of RDD controls had unlisted numbers [10]). Thus, RDD provides an ideal source when phone coverage is near complete; 93% of NYC residences have phones [11]. High phone coverage makes RDD one of the best sources for generating a sampling frame for controls of NYC area women under 65 years of age. Even when the source population is not solely defined by geography, a modified version of RDD is available that creates a control sampling frame using the cases' telephone numbers [10, 12]. This is the approach that was used in the WCHS in NY. RDD controls have been compared to a privately conducted census population [13] as well as to area survey controls [14], and both comparisons found that RDD controls were similar to those from other sources. Most importantly, high response rates within a minority community were demonstrated using the modified Waksberg RDD method [15], and in the WCHS, response rates among minorities are similar to those among EA women. The elimination of household landline phones in favor of cell phones represents a challenge for telephone surveys based on RDD to landline telephones [16, 17]. However, because the percentage of households without landlines remains low [17], any potential bias associated with this issue is likely to be small. Furthermore, once subjects agree to participate in the study, cell phones tend to facilitate scheduling interviews and completing study materials because the calls go directly to the participants and are not screened by other household members.

For RDD in NYC, the telephone exchanges (area code plus three-digit prefixes) of the breast cancer cases who received medical care at the participating hospitals in previous years were used for sampling. We frequency matched controls to cases on the expected breast cancer case distribution (based on 1994–1998 data from the NYS Tumor Registry) by 5-year age groups and race. The age distribution of targeted controls was periodically modified based upon the actual distributions of age among the cases. Controls were identified, recruited, and interviewed in the same manner and during the same time period as the cases to eliminate any bias related to secular trends or changes over the interviewing period.

In NJ, the same methodology is used for ascertainment of eligible controls; however, rather than using telephone numbers from participating hospitals, the entire county is sampled, because cases include those from all hospitals in the seven targeted counties. Controls, once identified, are contacted to schedule an in-person interview; interviews are conducted either at the participant's home or at another convenient location.

For both cases and controls in NYC and NJ who decline participation, we request that they complete a short telephone interview (5–10 minutes) to obtain basic information on demographic and exposure factors. In the final analysis, data from women who refused study participation will be compared to data from women who completed an interview to evaluate potential bias related to non-participation. Women who complete the study are offered a $50 gift certificate to one of several local stores as incentive for participation. We had initially offered $25 at the beginning of the study, but later increased the amount due to inflation and efforts to increase participation.

### 2.4. Data Collection—Interviews and Specimen Collection

The in-person interview consists of the informed consent process, an in-depth in-person interview, completion of several behavioral questionnaires including a Food Frequency Questionnaire (FFQ), collection of biospecimens, and body measurements. For cases, we also request a release for access to medical records, pathology data and for tumor tissue, as well as permission to conduct followup.

The survey instrument is an adaptation of several questionnaires, including validated surveys from the Women's Health Initiative and the Western New York Diet Study. Developmental history questions were taken from the Women's Interview Study of Health (WISH) [18], and lifetime physical activity is assessed using a modified version of Friedenreich's validated questionnaire [19]. Information on medical history, family history of cancer, lifestyle factors including smoking, alcohol consumption, and use of hair products is also collected. The most recent version of the FFQ developed at Fred Hutchinson Cancer Center and validated in the NCI/SWOG Prostate Cancer Prevention Trial is used for dietary assessment. This FFQ has been validated for use in an AA population. At the end of the visit, detailed measurements of current body size are taken. Participants are asked to wear light clothing, as weight, standing height, and waist, and hip circumferences are measured. Body composition (lean and fat mass) is measured using a bioelectrical impedance analysis scale (Tanita scale). Questionnaires are coded by two separate RAs, and double data entry is performed by two separate clerks, with data managed at RPCI.

Interviews take approximately 2 hours to complete, including anthropometry measures. We initially collected blood samples which were processed and stored in the laboratory at MSSM. In 2007, to reduce costs and to facilitate participation, we transitioned to collection of saliva using Oragene Kits (DNA Genotek, Inc, Ottawa, ON, Canada) for DNA extraction. These collection kits yield large quantities of high-quality DNA, comparable to that obtained from whole blood [20, 21].

Periodically, DNA has been extracted in batches, using the DNA Genotek Inc. protocol for DNA extraction from saliva or the FlexiGene method (Qiagen Inc, Valencia, CA) for whole blood or buffy coat. DNA is evaluated for purity and concentration using a Nanodrop UV spectrophotometer to obtain A230, A260, and A280 readings, and double stranded DNA is quantitated using a PicoGreen-based fluorometric assay (Molecular Probes, Invitrogen Inc, Carlsbad, CA). Saliva specimens have been stored at room temperature until extraction, and DNA samples are stored at −80°C at RPCI.

### 2.5. Collection of Tumor Tissue Blocks and Clinical Data

Formalin-fixed paraffin-embedded blocks and corresponding pathology reports from patients who signed the pathology and tissue release have been retrieved from hospitals on an ongoing basis. To date, 1193 patients have agreed for release of their tumor tissue (91%), and this proportion does not vary between NJ and NY. Pathology reports are reviewed in order to identify a representative tumor block used to make the primary breast cancer diagnosis for each case. The tumor blocks are shipped to RPCI, where they are labeled and entered into the tracking database. Hematoxylin and eosin (H&E) slides are cut and reviewed by the study pathologist (HH) to determine the locations from which cores should be taken for construction of tissue microarrays (TMAs), taking punches from both tumor and normal tissues and for consistent determination of grade by one pathologist. Representative tumor tissue is also labeled and punches taken to be stored for future DNA extraction and analysis. Pathology departments that do not release blocks have instead been asked to process and cut the requested number of slides (eleven unstained 5 *μ* slides and six unstained 10 *μ* slides), which are then sent to the laboratory at RPCI. Tissue blocks and pathology reports are collected in tandem and include the abstraction of medical record data. Because the consent process includes a tissue block and medical record release form, and blocks are being requested in “real time”, there has been little resistance on the part of the hospitals to provide tissue.

### 2.6. Challenges and Adaptations to Meet Them

In establishing the infrastructure for this study, and making efforts to conduct a study based in community hospitals in the face of stringent HIPAA and confidentiality requirements, our group brainstormed and adapted to achieve maximum case ascertainment, contact of patients, and recruitment into the study. With the help of committed and dedicated clinicians, this approach was successful at some hospitals, but not all. Clearly, it places a burden on already busy clinical practices, and it is likely that a complete denominator was not available, due to patients overlooked or deemed not suitable for participation in the study by their physician. In our experience, this is not a practical way to conduct a study and, unless one can ascertain cases through pathology reports or medical records, the costs of such efforts through local hospitals may not justify the numbers of cases able to be accrued. In contrast, by working through the NJDHSS, an NCI SEER site, we capture all cases diagnosed within a circumscribed area and truly know the denominator of the study for calculation of response rates. An additional advantage is that information on tumor characteristics is available for non-participating cases.

The trade-off is in participation rates. In NYC, when women were personally apprised of the study by their physician, response rates were relatively high, with 75% of EA and 75% of AA women completing interviews and providing blood or saliva samples. However, we have no data on the number of women who were eligible for the study and were not approached by their physician, or those who requested not to be contacted by our study staff.

When contacted by the NJDHSS, response rates are lower but still remain satisfactory. For EA women, 73% agreed to be contacted by an interviewer, and 93% of those women were interviewed and provided a saliva sample, for a total participation rate of 68%. Participation was poorer for AA women in NJ; 60% agreed to be contacted by an interviewer when telephoned by staff from the NJDHSS, and of those, 90% were enrolled into the study, for a total participation rate of 54%. We have met approximately half of our accrual goal, to date, and efforts are constantly made to improve response rates.

In NJ, the study is truly population-based. Newly diagnosed patients from all hospitals in the 7 targeted counties are ascertained and contacted by the NJDHSS. These counties provide the population to be captured by RDD as well. In NY, we focused on those hospitals with the highest referral patterns for AAs in the 5 boroughs excluding Staten Island, and it is clear that coverage was not complete. While an average of 1273 cases per year are reported in AA women in the boroughs, we were only able to ascertain approximately 67 per year through working with clinicians in selected hospitals. We expect that the control sampling frame in NY results in a representative population, nonetheless, because the first three numbers of breast cancer patients seen in previous years at each hospital were used to obtain women in the same residential areas.

When confronted with difficulties in case ascertainment in NYC, we sought ways to expand eligibility criteria without compromising the integrity of the study. We initially limited eligibility for case participants to those between the ages of 20 to 64 years, primarily because of the low response rates using RDD for controls 65 years and older. In 2007, we extended the upper limit of age eligibility to 75 years for cases, but not controls. Although these older women cannot be used in case-control comparisons, they will allow for case-case analysis of younger versus older age at onset of breast cancer, in which age of the patient is the dependent variable. This will allow us to explore possible differences in study variables (e.g., aggressive versus non aggressive disease characteristics) between older breast cancer patients and younger breast cancer patients. We will also explore the possibility that such differences might differ by race/ethnicity groups and by other disease characteristics defined by pathology.

We had initially trained WCHS interviewers in phlebotomy and made consent for specimen collection a requirement of the study. Three tubes of blood were collected and processed, with straws stored with plasma, serum, red blood cells (RBC), and buffy coat for DNA extraction. Our intent was, when possible, to collect pretreatment blood samples to be able to compare biomarkers in cases and controls and for use later in studies of breast cancer prognosis. Because of the difficulties in accrual in NYC, and in planning approaches in NJ where we knew that we would not be able to coordinate specimen collection prior to initiation of cancer therapy, we decided to collect saliva as a source of DNA only, using Oragene Saliva DNA Self-Collection Kits when we began recruitment in NJ. Again, our ideal approach would be to have pretreatment blood specimens on all cases, but in the interests of cost and feasibility and what was viewed as long term utility of samples other than DNA, compromises had to be made. To date, we have serum, plasma, and RBCs banked on 261 AA and 197 EA controls as well as 198 and 147 AA and EA cases, respectively, which should provide us with capabilities to investigate, in a limited sample set, differences in biomarkers among controls only, and case control evaluations for markers that are not likely to be affected by surgery or adjuvant therapy. All other cases and controls provided saliva samples, and there are no participants in the study for whom a source of DNA is not available.

## 3. Results

As noted above, case ascertainment and accrual in NYC was terminated in 2008, and all efforts are now ongoing and focused on enrollment in NJ. Table 1 shows current recruitment numbers for cases and controls, by race, in NYC and in NJ. For the scope of this paper, we are reporting data on the subset of cases and controls who have questionnaire data which have been processed and verified through double data entry, which includes 858 controls and 1119 cases. In examining preliminary data through February 2009, there are notable differences by race/ethnicity among participants. Because we are still in data collection phase, we have made limited comparisons between cases and controls in this report. Rather, we have contrasted demographic and tumor characteristics among AA and EA women in our study samples. Among controls (Table 2), there are differences in country of birth, with more AAs born in the Caribbean. EAs are more likely to be married, to have graduated college, and to have employer-provided health insurance. Higher proportions of EA women have incomes above $90,000 per year and EA women have fewer pregnancies and at a later age than AAs. Rates of screening mammography are similar between AA and EA women without breast cancer (86% and 87%, resp.). Notably, AA controls are more likely to be overweight than EAs (30% versus 25%) or obese (52% versus 26%) but are less likely to use hormone replacement therapy (HRT) than EAs (15% versus 24%).

Demographic characteristics of cases (Table 3) and differences by race/ancestry are, for the most part, similar to distributions for controls in terms of birthplace, marital status, education, health insurance, and income. Twenty percent of AA women with breast cancer in our study either do not have health insurance (17%) or pay for insurance out of pocket (3%), compared to 12% of EA cases (4% with no insurance, 8% self-purchased). In contrast to controls, where use of mammography is similar by race/ancestry, only 78% of AA cases ever had a screening mammography, compared to 88% of EA women, and 51% of EA cases had their breast cancer discovered by mammography versus only 36% of AA women. There also appear to be greater differences by race/ancestry for hormonal and reproductive factors among cases than among controls. Twenty-nine percent of AA cases experienced menarche at or below age 12, compared to only 24% of EA women; these differences are not as notable among controls (27% versus 25%). African American cases also tend to have more children and at an earlier age than EA cases, similar to patterns observed among controls. As observed for controls, AA women with breast cancer are also more likely to be overweight (31%) or obese (53%) than EA cases (26% and 26%, resp.) and are less likely to use HRT than EAs (15% versus 27%).

Of the pathology reports abstracted to date, the characteristics of tumors of women in our study are similar to those noted in literature [1]. African-American women are more likely than EA to have high-grade tumors (52% versus 32%) with ER negative (34% versus 22%) and PR negative (48% versus 34%) status. There are negligible differences by ancestry for HER2 status in our study population.

It is possible that differing methods of ascertainment and accrual could result in selection bias. We compared clinical and some epidemiological data between participants in NY and those in NJ. As shown in Table 4, AA cases from NY are more likely to have less than 11th grade education (22% versus 9%), more likely not to have health insurance (23% versus 9%), or be receiving Medicaid (21% versus 8%). Cases in NY had a lower incidence of DCIS (21% versus 13%), with invasive cancers being slightly higher (87% versus 79%). These differences may be due to the fact that, in New York, the majority of AA cases were ascertained at Kings County Hospital in Brooklyn which serves a large Caribbean community, many with low socioeconomic status, or because participation rates were higher in NY, resulting in some selection bias among those who agreed to be contacted in NJ.

For EA patients (Table 5), NY cases were more likely to be postgraduates (36% versus 22%) and but were less likely to have insurance (5% versus 2%) and receive Medicaid (4% versus 0%). Cases in NY were less likely to be obese (32% versus 22%) and had an older age at menarche (52% versus 42%).

Differences between controls in NY and NJ (Tables 6  and 7) showed some similar patterns as those for cases. NY AA controls were more likely to be on Medicaid (18% versus 10%) and were more likely to be obese (55% versus 34%). Similar differences were noted for EA controls.

It is difficult to ascertain the representativeness of our participants in relation to the underlying populations they were derived from. However, we did ask those who refused to be interviewed to complete a short telephone interview. In NY, cases who refused tended to be older >49, insured, either through Medicaid, Medicare, or employee-based insurance, have never taken hormone replacement therapy, and have had screening mammograms. Similar differences were noted for cases in NJ and for controls (insured, no HRT, and higher prevalence of screening mammograms). For controls, those who refused were more likely to have employer-provided insurance. The higher participation rates of cases in NY suggest that there would be less selection bias than in NJ, particularly for AA cases, because of lower participation rates in NJ. On the other hand, the population of cases in NY is somewhat skewed towards those treated at the County Hospital, where there is a large Caribbean population.

## 4. Discussion and Future Directions

When embarking on the conduct of a case-control study, a number of factors should be considered with respect to methodology. Uppermost in importance is feasibility, which is often overlooked by young, eager investigators. Although we recruited and interviewed over 500 cases through hospitals in NYC, the approach was often a struggle, and there is no question that case ascertainment through collaboration with a state SEER Cancer Registry is much more efficient. Using this approach, we are currently interviewing over 60 women per month, with numbers expected to rise with additional interviewers hired. We are confident that we will reach our accrual goals within the next 24 to 36 months, with ample power to evaluate our main study hypotheses, yielding important information regarding the etiology of aggressive breast cancers among AA as well as EA women. Since initiating the study, scientific knowledge has advanced, and while our earlier aims were to categorize women according to age at onset, tumor grade, and ER status, we are currently reclassifying tumor grade based on readings from one pathologist and building TMAs with funding from the Breast Cancer Research Foundation to stain and read all tissue for ER, PR, and HER2 for assessment of triple negative breast cancers as well as cytokeratins 5 and 6 and HER1 to help classify basal-like breast cancers. The successful enrollment of cases and controls, and collection of tissue blocks, has also facilitated numerous collaborations for pooled studies to conduct genomewide association studies and to determine the extent of African admixture in relation to tumor characteristics. With tumor tissue DNA as well as TMAs in addition to the epidemiologic data and biospecimens, we will have numerous opportunities not only to address our primary hypotheses but also to address novel hypotheses regarding ethnic/racial disparities in breast cancer incidence and mortality.

## 5. Conclusion

Epidemiological research has become increasingly difficult with the growing concerns regarding privacy and legal issues. To be able to address pressing issues in breast cancer research, particularly causal factors for the more aggressive breast cancers in AA women, creative strategies are required to conduct hospital and population-based studies. Partnership with SEER site is one approach for successful and complete case ascertainment and can facilitate the needed research in breast cancer disparities.

## Figures and Tables

**Table 1:** Distribution of study participants by race, state, and case/control status as of June 2009. Numbers in tables vary, subject to status of double data entry of questionnaires and receipt and entry of pathology reports.

	Cases (*n* = 1, 315)		Controls (*n* = 1, 097)	
	African American	European American	African American	European American
New York City	339	342	356	336
New Jersey	284	350	93	312
Total	623	692	449	648

**Table 2:** Characteristics of 858 controls.

	African American		European American	
	*N* (412)	%	*N* (446)	%
*Age at interview*				
* * <35	21	5.1	22	4.9
* *35–39	21	5.1	37	8.3
* *40–49	119	28.9	127	28.5
* *50–59	172	41.8	194	43.5
* *60–64	73	17.7	61	13.7
* *65+	6	1.5	5	1.1
*Country of origin* ^1^				
* *United States and Canada	280	68.0	390	87.4
* *Caribbean countries	63	15.3	0	0
* *Other	69	16.7	56	12.6
*Marital status* ^1^				
* *Married	143	34.9	277	62.1
* *Living as married	15	3.7	19	4.3
* *Widowed	21	5.1	17	3.8
* *Separated	34	8.3	15	3.4
* *Divorced	70	17.1	48	10.8
* *Single, never married or never lived as married	127	31.0	70	15.7
*Highest grade of school completed* ^1^				
* *Less than 11th grade	52	12.6	7	1.6
* *High school graduate or equivalent	91	22.1	30	6.7
* *Some college	128	31.1	87	19.5
* *College graduate	86	20.9	156	35.0
* *Post-graduate degree	55	13.4	166	37.2
*Health insurance (multiple choices possible)*				
* *Medicaid^1^	70	17.0	17	3.8
* *Medicare^1^	17	4.1	7	1.6
* *Employer-provided insurance^1^	272	66.2	350	78.5
* *Pay for insurance out of pocket^1^	18	4.4	49	11.0
* *I do not have health insurance	29	7.0	23	5.2
* *Other	13	3.2	21	4.7
*Annual income* ^1^				
* *Less than $15 000	51	13.4	15	3.6
* *$15 000–19 999	30	7.9	9	2.2
* *$20 000–24 999	25	6.5	5	1.2
* *$25 000–34 999	48	12.6	19	4.6
* *$35 000–49 999	68	17.8	42	10.2
* *$50 000–69 999	60	15.7	53	12.9
* *$70 000–89 999	45	11.8	61	14.8
* *$90 000 or more	55	14.4	208	50.5
*BMI* ^1^				
* *Underweight	3	0.8	14	3.5
* *Normal	68	17.7	188	46.3
* *Overweight	115	30.0	100	24.6
* *Obese	198	51.6	104	25.6
*Age at menarche*				
* * <11	40	9.7	39	8.8
* *11-12	71	17.3	69	15.6
* *12-13	90	21.9	112	25.3
* *13-14	99	24.1	124	28.0
* *14+	111	27.0	99	22.4
*Number of pregnancies* ^1^				
* *No pregnancies	36	9.9	91	23.4
* *1 pregnancy	89	24.4	90	23.1
* *2 pregnancies	103	28.2	126	32.4
* *3 pregnancies	74	20.3	56	14.4
* *4 pregnancies	31	8.5	8	2.1
* *5 + pregnancies	32	8.8	18	4.6
*Age at first pregnancy* ^1^				
* * ≤19	115	35.5	18	6.1
* *20–24	107	33.0	65	22.0
* *25–29	50	15.4	80	27.0
* *30+	52	16.1	133	44.9
*Age at menopause* ^1^				
* *Premenopausal	114	32.4	158	38.7
* *Perimenopausal	104	29.6	117	28.7
* * ≤44	26	7.4	13	3.2
* *45–49	44	12.5	31	7.6
* *50+	64	18.2	89	21.8
*Ever have hormone replacement therapy?* ^1^				
* *Yes	63	15.4	105	23.6
* *No	347	84.6	340	76.4
*Ever have a screening mammogram?*				
* *Yes	353	86.1	388	87.0
* *No	57	13.9	58	13.0

^1^ *P* < .05, Chi-square or Fisher's exact test, as appropriate, for differences between AAs and EAs.

**Table 3:** Characteristics of 1119 breast cancer cases.

	African American		European American	
	*N* (559)	%	*N* (560)	%
*Age at interview*				
* * <35	28	5.0	17	3.0
* *35–39	33	5.9	32	5.7
* *40–49	179	32.0	179	32.0
* *50–59	207	37.0	198	35.4
* *60–64	79	14.1	89	15.9
* *65+	33	5.9	44	7.9
*Country of origin* ^1^				
* *United States and Canada	338	60.5	472	84.3
* *Caribbean countries	130	23.2	8	1.4
* *Other	91	16.3	80	14.3
*Marital status* ^1^				
* *Married	195	35.1	354	63.5
* *Living as married	13	2.3	17	3.1
* *Widowed	37	6.7	23	4.1
* *Separated	49	8.8	10	1.8
* *Divorced	93	16.7	61	11.0
* *Single, never married or never lived as married	169	30.4	92	16.5
*Highest grade of school completed* ^1^				
* *Less than 11th grade	94	16.8	13	2.3
* *High school graduate or equivalent	155	27.7	81	14.5
* *Some college	160	28.6	120	21.5
* *College graduate	98	17.5	180	32.2
* *Post-graduate degree	52	9.3	165	29.5
*Health insurance (multiple choices possible)*				
* *Medicaid^1^	87	15.6	13	2.3
* *Medicare	39	7.0	31	5.6
* *Employer-provided insurance^1^	328	58.7	455	81.4
* *Pay for insurance out of pocket^1^	18	3.2	42	7.5
* *I do not have health insurance^1^	95	17.0	22	3.9
* *Other	19	3.4	20	3.6
*Annual income* ^1^				
* *Less than $15 000	103	20.9	25	4.9
* *$15 000–19 999	66	13.4	11	2.2
* *$20 000–24 999	37	7.5	12	2.4
* *$25 000–34 999	54	10.9	14	2.8
* *$35 000–49 999	66	13.4	50	9.9
* *$50 000–69 999	67	13.6	54	10.7
* *$70 000–89 999	39	7.9	68	13.4
* *$90 000 or more	62	12.6	273	53.9
*BMI* ^1^				
* *Underweight	6	1.2	9	1.8
* *Normal	78	15.5	239	46.4
* *Overweight	156	30.9	132	25.6
* *Obese	265	52.5	135	26.2
*Age at menarche* ^1^				
* * <11	72	12.9	48	8.7
* *11-12	87	15.6	83	15.0
* *12-13	120	21.5	160	28.9
* *13-14	132	23.7	146	26.3
* *14+	147	26.3	117	21.1
*Number of pregnancies* ^1^				
* *No pregnancies	43	8.4	117	23.9
* *1 pregnancy	112	21.8	101	20.7
* *2 pregnancies	161	31.4	166	33.9
* *3 pregnancies	94	18.3	68	13.9
* *4 pregnancies	51	9.9	24	4.9
* *5 + pregnancies	52	10.1	13	2.7
*Age at first pregnancy* ^1^				
* *≤19	172	37.1	27	7.3
* *20–24	140	30.2	93	25.0
* *25–29	88	19.0	110	29.6
* *30+	64	13.8	142	38.2
*Age at menopause* ^1^				
* *Premenopausal	200	43.4	207	40.8
* *Perimenopausal	115	24.9	97	19.1
* * ≤44	28	6.1	20	3.9
* *45–49	46	10.0	54	10.6
* *50+	72	15.6	130	25.6
*Ever have hormone replacement therapy?* ^1^				
* *Yes	82	14.8	152	27.2
* *No	473	85.2	406	72.8
*Ever have a screening mammogram?* ^1^				
* *Yes	435	78.0	492	88.2
* *No	123	22.0	66	11.8
*How was your breast cancer found?* ^1^				
* *Routine self-exam	144	26.0	63	11.4
* *Accidental self discovery	128	23.2	106	19.1
* *Accidental discovery by a partner	6	1.1	4	0.7
* *Routine physical exam by a doctor	37	6.7	42	7.6
* *Routine mammogram	198	35.8	283	51.0
* *Some other way	40	7.2	57	10.3
*ER status* ^1^				
* *Positive	231	65.6	203	77.8
* *Negative	121	34.4	58	22.2
*PR status* ^1^				
* *Positive	181	51.7	172	66.4
* *Negative	169	48.3	87	33.6
*HER2*				
* *Positive	83	27.7	41	20.8
* *Negative	217	72.3	156	79.2
*Grade* ^1^				
* *Well-differentiated	35	8.6	68	20.9
* *Moderately differentiated	162	39.8	153	47.1
* *Poorly differentiated	210	51.6	104	32.0

^1^ *P* < .05, Chi-square or Fisher's exact test, as appropriate, for differences between AAs and EAs.

**Table 4:** Characteristics of 559 African American breast cancer cases.

	New Jersey		New York	
	N (226)	%	N (333)	%
*Age at interview*				
* * <40	20	8.9	41	12.3
* *50–59	154	68.1	232	69.7
* *60+	52	23.0	60	18.0
*Highest grade of school completed* ^1^				
* *Less than 11th grade	20	8.9	74	22.2
* *High school graduate or equivalent	63	27.9	92	27.6
* *Some college	76	33.6	84	25.2
* *College graduate	43	19.0	55	16.5
* *Postgraduate degree	24	10.6	28	8.4
*Health insurance (multiple choices possible)*				
* *Medicaid^1^	18	8.0	69	20.7
* *Medicare	16	7.1	23	6.9
* *Employer-provided insurance^1^	169	74.8	159	47.8
* *Pay for insurance out of pocket	10	4.4	8	2.4
* *I do not have health insurance^1^	20	8.9	75	22.5
* *Other	10	4.4	9	2.7
*BMI*				
* *Underweight	1	0.5	5	1.6
* *Normal	28	14.1	50	16.3
* *Overweight	59	29.8	97	31.6
* *Obese	110	55.6	155	50.5
*First degree relative with breast cancer*				
* *Yes	37	16.4	45	13.5
* *No	189	83.6	288	86.5
*Age at menarche*				
* * <11	27	12.0	45	13.5
* *11–13	82	36.4	125	37.5
* *13+	116	51.6	163	49.0
*ER status*				
* *Positive	45	68.3	76	63.8
* *Negative	97	31.7	134	36.2
*Grade*				
* *Well-differentiated	15	8.2	20	8.9
* *Moderately differentiated	72	39.3	90	40.0
* *Poorly differentiated	96	52.5	115	51.1
*Histologic type* ^1^				
* *DCIS	43	20.8	30	13.0
* *Invasive	164	79.2	201	87.0

^1^ *P* < .05, Chi-square or Fisher's exact test, as appropriate, for differences between states.

**Table 5:** Characteristics of 560 European American breast cancer cases.

	New Jersey		New York	
	*N* (252)	%	*N* (308)	%
*Age at interview*				
* * <40	18	7.1	31	10.1
* *50–59	166	65.9	211	68.7
* *60+	68	27.0	65	21.2
*Highest grade of school completed* ^1^				
* *Less than 11th grade	6	2.4	7	2.3
* *High school graduate or equivalent	47	18.7	34	11.1
* *Some college	60	23.8	60	19.5
* *College graduate	83	32.9	97	31.6
* *Postgraduate degree	56	22.2	109	35.5
*Health insurance (multiple choices possible)*				
* *Medicaid^1^	0	0.0	13	4.2
* *Medicare	15	6.0	16	5.2
* *Employer-provided insurance^1^	220	87.7	235	76.3
* *Pay for insurance out of pocket	17	6.8	25	8.1
* *I do not have health insurance	6	2.4	16	5.2
* *Other	9	3.6	11	3.6
*BMI* ^1^				
* *Underweight	2	0.9	7	2.5
* *Normal	98	42.6	141	49.5
* *Overweight	57	24.8	75	26.3
* *Obese	73	31.7	62	21.8
*First degree relative with breast cancer*				
* *Yes	58	23.0	83	27.0
* *No	194	77.0	225	73.0
*Age at menarche* ^1^				
* * <11	25	10.0	23	7.5
* *11–13	120	48.2	123	40.3
* *13+	104	41.8	159	52.1
*ER status* ^1^				
* *Positive	109	77.9	28	77.4
* *Negative	31	22.1	96	22.6
*Grade*				
* *Well-differentiated	43	22.8	25	18.1
* *Moderately differentiated	88	46.6	65	47.1
* *Poorly differentiated	58	30.7	48	34.8
*Histologic type*				
* *DCIS	56	25.2	40	27.6
* *Invasive	166	74.8	105	72.4

^1^ *P* < .05, Chi-square or Fisher's exact test, as appropriate, for differences between states.

**Table 6:** Characteristics of 412 African American controls.

	New Jersey		New York	
	*N* (63)	%	*N* (349)	%
*Age at interview*				
* * <40	11	17.5	30	8.6
* *40–59	40	63.5	252	72.2
* *60+	12	19.0	67	19.2
*Highest grade of school completed* ^1^				
* *Less than 11th grade	7	11.1	45	12.9
* *High school graduate or equivalent	13	20.6	77	22.1
* *Some college	18	28.6	110	31.5
* *College graduate	15	23.8	71	20.3
* *Postgraduate degree	10	15.9	46	13.2
*Health insurance (multiple choices possible)*				
* *Medicaid	6	9.5	63	18.1
* *Medicare	3	4.8	14	4.0
* *Employer-provided insurance	43	69.4	230	65.9
* *Pay for insurance out of pocket	3	4.8	15	4.3
* *I do not have health insurance^1^	4	6.4	25	7.2
* *Other^1^	5	7.9	8	2.3
*BMI* ^1^				
* *Underweight	0	0.0	2	0.6
* *Normal	15	26.8	54	16.5
* *Overweight	22	39.3	93	28.4
* *Obese	19	33.9	179	54.6
*First degree relative with breast cancer*				
* *Yes	5	7.9	34	9.7
* *No	58	92.1	315	90.3
*Age at menarche*				
* * <11	6	9.5	35	10.1
* *11–13	26	41.3	135	38.8
* *13+	31	49.2	178	51.2

^1^ *P* < .05, Chi-square or Fisher's exact test, as appropriate, for differences between states.

**Table 7:** Characteristics of 446 European American controls.

	New Jersey		New York	
	*N* (124)	%	*N* (322)	%
*Age at interview* ^1^				
* * <40	23	18.6	36	11.2
* *40–59	89	71.8	232	72.1
* *60+	12	9.7	54	16.8
*Highest grade of school completed*				
* *Less than 11th grade	1	0.8	6	1.9
* *High school graduate or equivalent	8	6.5	21	6.5
* *Some college	31	25.2	56	17.4
* *College graduate	44	35.8	112	34.8
* *Post-graduate degree	39	31.7	127	39.4
*Health insurance (multiple choices possible)*				
* *Medicaid	2	1.6	15	4.7
* *Medicare	1	0.8	6	1.9
* *Employer-provided insurance^1^	105	85.4	244	75.8
* *Pay for insurance out of pocket	12	9.7	37	11.5
* *I do not have health insurance	6	4.8	17	5.3
* *Other	3	2.4	18	5.6
*BMI*				
* *Underweight	1	0.8	13	4.5
* *Normal	48	41.0	140	48.6
* *Overweight	34	29.1	65	22.6
* *Obese	34	29.1	70	24.3
*First degree relative with breast cancer*				
* *Yes	15	12.1	49	15.2
* *No	109	87.9	273	84.8
*Age at menarche*				
* * <11	14	11.5	25	7.8
* *11–13	50	41.0	131	40.9
* *13+	58	47.5	164	51.3

^1^ *P* < .05, Chi-square or Fisher's exact test, as appropriate, for differences between states.
